# Glabrol—an impurity in licorice extract—causes toxicity in muscle, bone, and immune tissues through activation of the AP-1 signaling pathway

**DOI:** 10.3389/fphar.2026.1795069

**Published:** 2026-03-27

**Authors:** Chenyang Jiang, Xinshu Feng, Mingyu Qi, Shaojuan Wu, Qiuyu Wang, Yawen Zhou, Xiaoshan Zheng, Zhuogui Liang, Jianhua Tan, Yudong Zhang, Qingquan Guo, Haishan Zhao

**Affiliations:** 1 Guangdong Provincial Key Laboratory of South China Structural Heart Disease, Guangdong Cardiovascular Institute, Department of Medical Research, Guangdong Provincial People’s Hospital, Guangdong Academy of Medical Sciences, Southern Medical University, Guangzhou, China; 2 Guangzhou Shanhai Biotechnology Co., Ltd., Guangzhou, China; 3 Guangzhou Tongjun Pharmaceutical Technology Co., Ltd., Guangzhou, China; 4 Department of Allergy and Clinical Immunology, National Clinical Research Center for Respiratory Disease, State Key Laboratory of Respiratory Disease, Guangzhou Institute of Respiratory Health, The First Affiliated Hospital of Guangzhou Medical University, Guangzhou, China; 5 Guangdong Provincial Key Laboratory of South China Structural Heart Disease, Guangdong Cardiovascular Institute, Guangdong Provincial People’s Hospital (Guangdong Academy of Medical Sciences), South China University of Technology, Guangzhou, China; 6 Guangzhou Quality Supervision and Testing Institute, Guangzhou, China; 7 School of Light Chemical Engineering, Guangdong University of Technology, Guangzhou, China

**Keywords:** AP-1 signaling pathway, biological effects, glabrol, licorice, toxicity

## Abstract

Licorice (*Glycyrrhiza genus*) is a traditional medicinal herb that has also been widely used in the food and cosmetic industries, leading to widespread human exposure. Currently, many components have been identified as active ingredients in licorice; however, the toxic impurities and quality markers still require further investigation. Glabrol has been identified as a potentially toxic component in glabridin (an extract from licorice). In this study, we sought to evaluate the toxicity of glabrol in commercial licorice extracts and investigated the toxicological mechanism. The content of glabrol and the acute toxicity in ten commercial licorice extracts from different vendors were quantified using HPLC. The toxicity was further verified in zebrafish, cells *in vitro* and mammals *in vivo mouse models*. For the *in vivo* experiments, C57BL/6 mice received daily oral gavage of licorice extracts with (Sample C: 319.23 μg/g) o low (Sample B: 1.54 μg/g) glabrol content for 7 days. Locomotion was assessed *via* Open Field Test and Elevated Plus Maze, followed by blood and organ collection for pathological and biochemical analyses. To investigate the toxicological mechanism of glabrol, RNA - seq was performed on zebrafish embryos exposed to glabrol. Morphological and histopathological evaluations in zebrafish treated with the glabrol standard were carried out using phalloidin staining, transmission electron microscopy, and alizarin red staining. Our results indicated that glabrol was detected in all tested commercial licorice extracts, and its content showed a significant positive correlation with toxicity in cells and zebrafish. In mice, licorice extracts with higher glabrol levels led to low survival rates, hypoactivity, acute liver and kidney injury, and significantly elevated plasma inflammatory cytokines. Transcriptomic and mechanistic studies revealed that glabrol disrupted AP-1 signaling pathways and may impair myo-fiber organization, osteoclast differentiation, and inflammatory responses. This study establishes glabrol as a prevalent risk-associated impurity in licorice extracts and reveals that its toxicity is mediated *via* the AP-1 signaling pathway.

## Graphical Abstract

**Figure:**
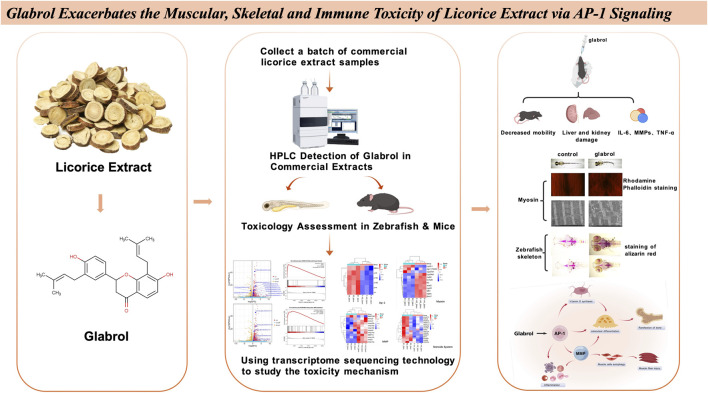

## 1 Introduction

Licorice (*Glycyrrhiza genus*), an important herb in the Leguminosae family with a medicinal history of approximately 4,000 years (Guo et al., 2022), exhibits broad pharmacological activities due to its multi-target active components. In medicine, its extracts are utilized for their anti-inflammatory, antiviral, hepatoprotective, immunomodulatory, anti-ulcer, and anti-tumor effects (El-Saber Batiha et al., 2020; Yu et al., 2022), and are also employed in traditional Chinese medicine. In the food industry, it serves as a functional sweetener in products such as preserved fruits, candy, chewing gum, biscuits, and canned meats. In cosmetics, licorice extract is primarily used for its skin-lightening and spot-removing properties (Liu J. et al., 2025). Collectively, the historical and versatile bioactive profile of licorice underpins its wide-ranging applications across pharmaceutical, food, and cosmetic sectors.

Although licorice is generally considered clinically safe, its widespread use in consumer products leads to substantial daily exposure among both healthy individuals and patients. Emerging evidence, however, has reported several adverse effects associated with licorice. Clinical observations indicate that high-dose intake can induce hypertension, hypokalemia, and even acute intoxication, primarily mediated by a mineralocorticoid effect. Animal studies further suggest dose-dependent toxicities, including renal injury, embryonic developmental malformations, and bradycardia, along with mild irritancy to the ocular conjunctiva (Caré et al., 2023). Since the risks may be associated with multiple impurities, quality control is essential for licorice extracts.

Currently, the quality assessment of licorice primarily relies on physical characteristics and the quantification of specific known compounds, such as glycyrrhizic acid and liquiritin, as stipulated by the United States, European, Japanese, and Chinese Pharmacopoeias (United States Pharmacopeial Convention; European Directorate for the Qualit y of Medicines and Healthcare; Ministry of He alth and Labour and Welfare (Japan); Chinese Pharmacopoeia Commission, 2020). However, these characterized constituents represent only a minor fraction of the total extract composition, leaving the biological roles and safety implications of numerous unknown components largely uninvestigated. Consequently, conventional methods fall short in providing a comprehensive quality profile, particularly when dealing with non-characteristic peaks or low-abundance compounds.

Glabrol, a flavonoid compound in licorice extracts, has been identified in our previous studies as a key risk impurity that enhances the toxicity of glabridin - containing products (Guo et al., 2022). However, it remains unclear whether glabrol is universally present in common licorice extracts, and its underlying toxicological mechanisms have not been elucidated. Furthermore, current quality control standards for commercial licorice extracts do not include the monitoring of glabrol levels, leaving its potential impact on overall product safety largely unknown.

Therefore, this study aims to elucidate the toxicity mechanisms of glabrol through the following approaches: 1) Collect market-sourced licorice-extract lots, quantify glabrol content, and screen the acute toxicity in zebrafish embryos *in vivo* and cell lines *in vitro*, 2) use a mouse model to verify the *in vivo* toxicity of the licorice extract; 3) Dissect the underlying mechanism by RNA-seq profiling and zebrafish assays.

## 2 Materials and methods

### 2.1 Cell lines and reagents

The HepG2 and HepaRG cell lines were kindly provided by the research group of Professor Shi-Long Zhong, Department of Pharmacy, Guangdong Provincial People’s Hospital. Dulbecco’s Modified Eagle Medium (DMEM) and Endothelial Cell Medium (ECM) were obtained from ScienCell Research Laboratories (Carlsbad, CA, United States). Rhodamine Phalloidin reagent (Cat. No. CA1610) was sourced from Solarbio (Beijing, China). The Cell Counting Kit-8 (CCK-8) was acquired from TransGen Biotech (Beijing, China). Triton X-100 was supplied by Biofroxx (Einhausen, Germany). Alizarin Red S staining solution (0.2%, pH 8.3) was purchased from Beyotime Biotechnology (Shanghai, China). Bovine serum albumin (BSA, whole fraction) and glutaraldehyde fixative (4%, electron microscopy grade) were obtained from Solarbio (Beijing, China). HPLC-grade acetic acid (>99% purity) was provided by Aladdin (Shanghai, China). The glabrol reference standard (>98% purity) was acquired from Shanghai Yuanye Bio-Technology Co., Ltd. (Shanghai, China). Acetonitrile and formic acid of mass spectrometry grade (>99%) were sourced from Thermo Fisher Scientific (Guangzhou, China).

### 2.2 Sample collection

Ten licorice extract samples were randomly selected from Chinese e-commerce platforms Taobao and Alibaba. The samples were sequentially labeled A through J based on the order of purchase. All samples were from different manufacturers.

### 2.3 High-performance liquid chromatography (HPLC) assay

High-performance liquid chromatography (HPLC) analysis was carried out using a Waters Alliance system fitted with an ACQUITY UPLC BEH C18 column (100 mm × 2.1 mm, 1.7 μm; Agilent). The mobile phase, consisting of 10 mmol/L ammonium acetate in water containing 0.1% formic acid and methanol containing 0.1% formic acid (1:1, v/v), was delivered under isocratic conditions at a flow rate of 0.3 mL/min and a column temperature of 30 °C. A 2 μL aliquot of each sample was injected, and analytes were monitored at a wavelength of 534 nm. Quantification was performed using a glabrol reference standard, and all samples were analyzed in triplicate.

### 2.4 Zebrafish maintenance

The zebrafish were raised in the zebrafish facility of Guangzhou Shanhai Technology Biology Co., Ltd. The evening before the experiment, adult broodstock were placed into mating tanks at a 1:1 female-to-male ratio. The following morning, immediately after lights-on, the partition separating the sexes was removed to allow natural spawning. Fertilized eggs deposited within 30 min were collected using a fine mesh net, transferred to Petri dishes, and visually inspected. Viable embryos were then selected and incubated at 28 °C–29 °C in a temperature-controlled incubator.

### 2.5 Zebrafish embryo acute toxicity test

All zebrafish experiments were carried out in compliance with OECD Test Guidelines 203 and 236 and approved by the Ethics Committee of Guangdong Provincial People’s Hospital [Ethical Approval Number: (KY-Z-2022-204-01)]. At 24 h post-fertilization (hpf), healthy embryos were selected, rinsed, and examined under a Stemi 2000 stereomicroscope (Carl Zeiss, Jena, Germany). Individual embryos were transferred into 96-well plates (one per well) containing varying concentrations of glabrol or licorice extracts diluted in Holt buffer, with 12 biological replicates per treatment group. Based on the results of preliminary experiments, stock solutions of licorice extracts were prepared in Holt buffer (25 mg/mL) and serially diluted to yield final concentrations of 1,000–8,000 μg/mL for samples A, C, D, H, and E, and 5,000–25,000 μg/mL for other samples (dissolved in Holt buffer). The control group received Holt buffer. Holt buffer (60 mM NaCl, 0.67 mM KCl, 0.3 mM NaHCO_3_, 0.9 mM CaCl_2_) was prepared as previously described. Embryos were incubated at 28.0 °C–29.0 °C, and survival was assessed at 48, 72, and 96 hpf based on the presence of a heartbeat; cessation of cardiac activity was defined as death. Each experiment was independently repeated three times. Survival rates were calculated as: Survival rate (%) = (Number of live embryos/Total number of embryos per group) × 100.

### 2.6 Phenotype recording

Zebrafish embryos (24 hpf) were collected, randomly divided into groups (20 embryos per group), and continuously exposed to 1% DMSO or glabrol standard solutions (1, 2, and 3 µM). All embryos were cultured in 24-well cell culture plates (2 mL solution per well). At 48 hpf, embryos were individually photographed for phenotypic assessment using a stereomicroscope (Optec, Chongqing, China).

### 2.7 Cell culture

HepG2 cells were maintained in Dulbecco’s Modified Eagle Medium (DMEM; Sigma-Aldrich, St. Louis, MO, United States) supplemented with 10% fetal bovine serum (FBS; Biosolutions International, Melbourne, Australia), 100 U/mL penicillin, and 100 μg/mL streptomycin. The human HepaRG cell line was grown under the same basal medium, additionally containing 2 mmol/L L-glutamine and 10% heat-inactivated FBS, and incubated at 37 °C in a humidified 5% CO_2_ atmosphere.

### 2.8 Cytotoxicity assay

Guided by the acute toxicity data obtained from the zebrafish model, cells were seeded in 96-well plates (triplicate wells per group) and exposed to test samples (dissolved in PBS) at concentrations of 2–12 mg/mL (2, 4, 6, 8, 10, and 12 mg/mL) for 24 h. Subsequently, 10 µL of CCK-8 reagent was added to each well, followed by a 2-h incubation. Absorbance was recorded at 534 nm. All assays were independently repeated three times.

### 2.9 C57BL/6 mouse acute toxicity experiment

Wild-type C57BL/6J mice (6–8 weeks old) were sourced from Guangzhou Daoke Biomedical Co., Ltd. (Guangzhou, China). After a 24-h acclimatization period, animals were randomly assigned to the following groups (*n* = 5 per concentration): (1) vehicle control (0.9% physiological saline); (2) high-glabrol licorice extract (sample C) and low-glabrol licorice extract (sample B) at 0.4, 0.5, and 0.6 mg/mL, dissolved in 0.9% saline. Mice were housed under standard laboratory conditions (12-h light/dark cycle, 22 °C–24 °C, with *ad libitum* access to food and water). All treatments were administered once daily *via* oral gavage at a volume of 0.2 mL per 10 g body weight for seven consecutive days. The vehicle control group received normal saline, while the treatment groups received the corresponding licorice extract solutions. General health and behavior were monitored daily throughout the study. All experimental procedures were approved by the Ethics Committee of Guangdong Provincial People’s Hospital (Approval No. KY-Z-2022-204-01) and conducted in accordance with the NIH Guide for the Care and Use of Laboratory Animals.

### 2.10 Open field test (OFT) and elevated plus maze (EPM)

Mice from Section 2.9 were subjected to behavioral tests half an hour after gavage. The OFT apparatus consisted of a square open field (40 cm × 40 cm, 30 cm high) with a blue base. Each mouse was placed individually in one corner of the apparatus, and its activity was monitored and recorded for 10 min using the SMART™ tracking program. After each test, the arena was thoroughly cleaned with 75% ethanol to remove any odor cues that might affect mouse behavior. The EPM apparatus consisted of a plus-shaped maze with two open arms (50 × 10 cm), two enclosed arms (50 × 10 cm), and a central area (10 × 10 cm). The height of the EPM was 50 cm. Each mouse was placed in the central area, and its exploration track was recorded for 5 minutes. The SMART tracking program was used to monitor and record the time spent in the open vs. enclosed arms.

### 2.11 C57BL/6 mouse sample collection

At the desired time points, animals were anesthetized by intraperitoneal injection of sodium pentobarbital (80 mg/kg) and euthanized. Serum, heart, lung, liver, brain, and kidney were collected. Tissues for histological evaluation were fixed in 4% formaldehyde in phosphate-buffered saline, while other tissues were stored at −80 °C until further use.

### 2.12 Tissue sectioning and hematoxylin and eosin (H&E) staining

Tissues obtained from mice were first fixed in 4% paraformaldehyde solution overnight, then dehydrated through a graded alcohol series (70%, 80%, 90%, 95%, 100% I, and 100% II) for 15 min each, cleared in xylene, and embedded in paraffin. After trimming, tissue sections were cut into 5 µm thickness, stained with H&E solution, observed, and photographed under a microscope (Leica M205, Germany).

### 2.13 RNA isolation, cDNA preparation, and RNA sequencing (RNA-seq)

Total RNA was isolated from zebrafish embryos at 48 hpf using a Total RNA Column Extraction Kit (manufacturer’s protocol). RNA integrity and purity were evaluated by Agilent 2100 Bioanalyzer, agarose gel electrophoresis, and Nanodrop spectrophotometry. First-strand cDNA was synthesized from fragmented mRNA using M-MuLV reverse transcriptase and random hexamer primers, followed by RNA template degradation with RNase H. Second-strand cDNA was then generated using DNA Polymerase I and dNTPs. The resulting double-stranded cDNA was purified and subjected to end repair, A-tailing, and adapter ligation to construct sequencing libraries. Each library was prepared in triplicate and sequenced on an Illumina HiSeq™ 2500/4000 platform using 150 bp paired-end reads. Library preparation and sequencing were performed by Gene Denovo Biotechnology Co., Ltd. (Guangzhou, China). Raw reads were processed through quality filtering, and downstream analyses—including differential gene expression, Gene Ontology (GO) enrichment, and KEGG pathway enrichment—were conducted using the Omicsmart platform (https://www.omicsmart.com/).

### 2.14 Real-time quantitative polymerase chain reaction (RT-qPCR)

Total RNA was extracted from zebrafish embryos using the FastPure Cell/Tissue Total RNA Isolation Kit (Vazyme Biotech, Nanjing, China) and treated with DNase I to eliminate genomic DNA contamination. RNA purity was assessed with a NanoDrop 2000 spectrophotometer (Thermo Fisher Scientific, United States). One microgram of total RNA was reverse-transcribed into cDNA using PrimeScript™ RT Master Mix (TaKaRa, Cat. No. RR036A) under the following conditions: 37 °C for 15 min, followed by 85 °C for 5 s. Quantitative real-time PCR (qPCR) was performed on a Bio-Rad CFX96 Touch™ system. Each 20 µL reaction comprised 10 μL TB Green® Premix Ex Taq™ II (TaKaRa, RR820A), 0.8 µL each of 10 µM forward and reverse primers, 2 µL cDNA template, and 6.4 µL nuclease-free water. Primer sequences are listed in Table 1. All primer pairs showed amplification efficiencies of 95%–105% and correlation coefficients (*R* ^2^) > 0.999. Gene expression was normalized to β-actin and calculated using the 2^−^ΔΔCt method. Amplification was carried out with an initial denaturation at 95 °C for 10 min, followed by 40 cycles of 95 °C for 15 s and 60 °C for 1 min. Specificity was confirmed by melt curve analysis. Data represent mean ± SD from at least three independent biological replicates. Statistical differences among groups were evaluated by one-way ANOVA, with *p* < 0.05 considered significant.

**TABLE 1:** Ten commercially available glycyrrhiza extracts.

Sample	Manufacturer	Extraction ratio
A	Shanghai Yuanye Bio-Technology Co., Ltd.	10:1
B	Xi’an Quan’ao Biotechnology Co., Ltd.	10:1
C	Rhawn(Shanghai Macklin Biochemical Co., Ltd.)	10:1
D	Aladdin (Shanghai Macklin Biochemical Co., Ltd.)	10:1
E	Xi’an Shuang’aichi Biotechnology Co., Ltd.	10:1
F	Xi’an Xinlu Biotechnology Co., Ltd.	10:1
G	Xi’an Xinlu Biotechnology Co., Ltd.	20:1
H	Shaanxi Kanghe Biotechnology Co., Ltd.	10:1
J	Shaanxi New Tianyu Biotechnology Co., Ltd.	10:1

### 2.15 Zebrafish embryo exposure to glabrol standard

To evaluate the specific toxicological profile of glabrol, a high-purity standard (99%) was employed for zebrafish embryo exposure. Healthy embryos at 24 h post-fertilization (hpf) were randomly allocated into 6-well plates at a density of 20 embryos per well. The concentration range was determined *via* preliminary dose-finding assays: concentrations above 4 μm resulted in high mortality, while 1–3 μm induced clear developmental abnormalities without mass lethality. Consequently, 0.5 μm was established as the maximum non-toxic dose (NOAEL), and the 1–3 μm range was selected to characterize the specific toxicological phenotypes of glabrol. The glabrol standard was initially dissolved in DMSO to prepare a stock solution and subsequently diluted with standard embryo medium to reach the target exposure levels. To prevent solvent-induced interference, the final DMSO concentration in all groups was strictly maintained below 0.1% (v/v), with an equivalent concentration used for the vehicle control. All embryos were incubated at 28.5 °C ± 0.5 °C under a 14 h light/10 h dark photoperiod.

### 2.16 Rhodamine phalloidin staining

F-actin was labeled with Rhodamine Phalloidin following the manufacturer’s protocol. Briefly, at 48 hpf, 12 zebrafish embryos per group treated with the glabrol standard were collected and washed thrice with phosphate-buffered saline (PBS), then fixed in 200 µL of 4% formaldehyde (in PBS) at 25 °C–28 °C for 2 h. After removal of the fixative, embryos were rinsed three times with PBS, permeabilized with 100 µL of 0.1% Triton X-100 in PBS for 30 min, and again washed thoroughly with PBS. Subsequently, embryos were incubated with 100 µL of 1× Rhodamine Phalloidin working solution at 25 °C–28 °C for 60 min, followed by fluorescence imaging at an excitation wavelength of 546 nm.

### 2.17 Alizarin red staining

Alizarin Red S staining was employed to evaluate bone mineralization, as the dye selectively binds to calcium deposits in mineralized tissues. At 5 days post-fertilization (dpf), zebrafish larvae treated with the glabrol standard were anesthetized with 70 mg/L tricaine, rinsed twice with 1 mL embryo medium, and fixed in 4% paraformaldehyde (PFA) at room temperature for 2 h. Following fixation, samples were washed twice with 1× PBS (5 min each) and incubated overnight in the dark with 0.005% Alizarin Red S solution. Excess stain was removed by bleaching in a 1:1 mixture of 3% H_2_O_2_ and 2% KOH (final concentrations: 1.5% H_2_O_2_, 1% KOH) for 30 min. Cleared larvae were stored in 50% glycerol and imaged using a stereomicroscope. Mineralized areas were quantified using ImageJ software.

### 2.18 Transmission electron microscopy (TEM) analysis

Transmission electron microscopy (TEM) was used to examine the ultrastructure of skeletal muscle in zebrafish embryos. At 48 hpf, embryos from Section 2.16 were collected and initially fixed in 1 mL of 2.5% glutaraldehyde at 4 °C for 4 h. After three 15-min washes with PBS (pH 7.4), samples were post-fixed in 1% osmium tetroxide (in the dark, room temperature, 2 h), followed by another three PBS washes under identical conditions. Dehydration was performed through a graded ethanol series (30%–100%, 20 min per step), followed by two 15-min rinses in 100% acetone. Samples were then infiltrated with resin, embedded, and polymerized overnight. Ultrathin sections (60 nm) were cut using an ultramicrotome, mounted on formvar-coated 150-mesh copper grids, and double-stained with uranyl acetate and lead citrate. Grids were air-dried overnight at room temperature and imaged using a transmission electron microscope (Serve, Guangzhou).

### 2.19 Statistical analysis

All statistical analyses were performed using GraphPad Prism (version 9.0; GraphPad Software, San Diego, CA, United States). Survival curves were generated in OriginPro (OriginLab, Northampton, MA, United States). The half-lethal concentration (LC_50_) and corresponding 95% confidence intervals for test compounds were estimated by logistic regression. Comparisons between two groups were conducted using Student’s t-test, while differences among multiple groups were evaluated by one-way ANOVA. A *p* value <0.05 was considered statistically significant.

## 3 Results

### 3.1 Quantitative analysis of glabrol content in samples

**FIGURE 1:**
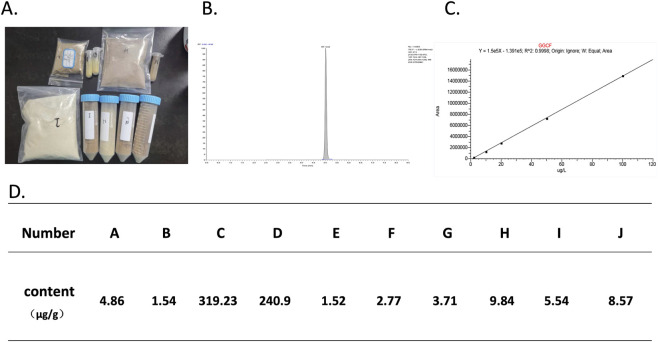
Glabrol was present to varying degrees in all commercial licorice extracts. **(A)** Commercially available licorice extracts from ten different brands (labeled A-J) were collected. **(B)** Chromatogram of the glabrol standard. **(C)** Standard curve for the quantification of glabrol in licorice extracts. **(D)** Actual glabrol content in licorice extracts from different brands. The highest glabrol content was observed in sample C (319.23 μg/g), while sample E exhibited the lowest content (1.52 μg/g). Notably, samples C and D showed significantly higher glabrol levels—by two orders of magnitude—compared to the other eight samples.

A total of ten licorice extract samples were collected. Preliminary judgment based on their varying color and properties suggested differences in composition among them (Figure 1A). First, quantitative analysis of glabrol content in the ten collected licorice extract samples was performed based on the standard curve established by HPLC (Figures 1B,C). The results showed that glabrol was present in all licorice extracts (Figure 1D). Sample C had the highest glabrol content at 319.23 μg/g, while Sample E had the least, only 1.52 μg/g, representing a difference of two orders of magnitude.

### 3.2 Acute toxicity of licorice extract samples on zebrafish embryos

**FIGURE 2:**
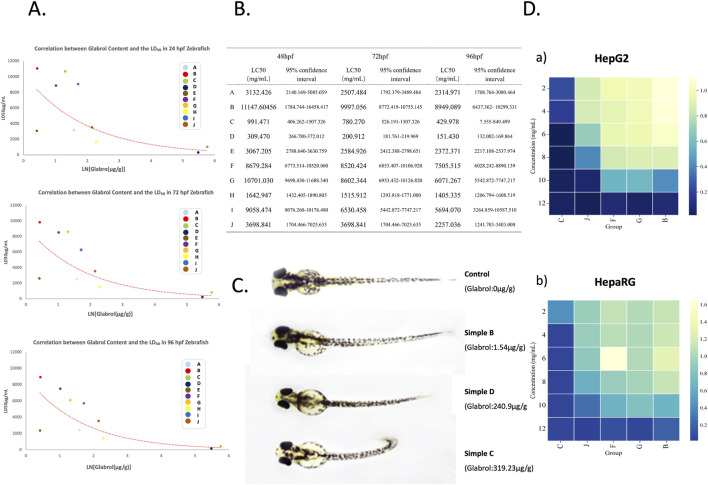
Evaluation of glabrol toxicity using *in vivo* and *in vitro* models. **(A,B)** The relationship between the LD_50_ in zebrafish at different developmental stages (48, 72, 96 hpf) and the glabrol content in the licorice extracts was investigated. **(C)** Treatment with extracts of varying glabrol concentrations induced significant morphological alterations in the somites of zebrafish larvae, which became progressively more severe with increasing glabrol content. **(D)** Cytotoxicity of glabrol standard was evaluated in **a** HepG2 and **b** HepaRG cell lines (unit: mg/mL).

To assess the *in vivo* safety of the samples, their toxicity towards zebrafish embryos was investigated using different concentrations of licorice extract samples (Samples C, D, H, E, A: 1, 2, 3, 4, 5, 6, 7, 8 mg/mL and Samples B, F, G, J, I: 5, 10, 15, 20, 25 mg/mL) based on the actual measured glabrol content. Cumulative mortality was recorded at 48, 72, and 96 hpf. Differences in biosafety were observed among the licorice extracts, with the actual glabrol content showing a generally negative correlation trend with the LD_50_ of the licorice extracts at 48, 72, and 96 hpf. The safety of licorice extracts decreased as the glabrol content increased. The two samples with high glabrol content, C (content: 319.23 μg/g) and D (content: 240.9 μg/g), exhibited relatively lower biosafety. The LC_50_ for Sample C was 991.471 μg/mL (48 hpf), 780.270 μg/mL (72 hpf), and 429.978 μg/mL (96 hpf). The LC_50_ for Sample D was 309.381 μg/mL (48 hpf), 200.818 μg/mL (72 hpf), and 150.89 μg/mL (96 hpf) (Figures 2A,B). Furthermore, significant changes in somite morphology were observed in zebrafish after administration of licorice extracts, and this was closely related to the glabrol concentration. The degree of somite curvature in zebrafish gradually intensified with increasing glabrol content in the licorice extracts (Figure 2C). Therefore, among the ten licorice extract samples, Samples C and D were highly toxic; Samples E, A, J, H were moderately toxic; and Samples B, F, G were low in toxicity.

### 3.3 Cytotoxicity of licorice extracts

To validate the reliability of the data obtained from the zebrafish model, the cytotoxicity of Samples B (1.54 μg/g), C (319.23 μg/g), F (2.77 μg/g), G (3.71 μg/g), and J (8.57 μg/g) on Human Hepatocellular Carcinoma G2(HepG2)and Hepatoma Re-Generating (HepaRG) cells was determined using the Cell Counting Kit-8(CCK-8 assay) (Figure 2D). As shown in the figures, Samples B, G, and F showed similar cytotoxicity towards both cell lines, with no significant inhibitory activity at 8 mg/mL. Sample J was slightly more toxic but showed no significant inhibition at 4 mg/mL. Notably, Sample C, with the highest glabrol content, exhibited far greater cytotoxicity than the other samples. These results confirmed the data obtained from the zebrafish experiments; therefore, we consider the results obtained using the zebrafish model to be reliable.

### 3.4 Toxicity of licorice extract samples in mammals and behavioral effects

**FIGURE 3:**
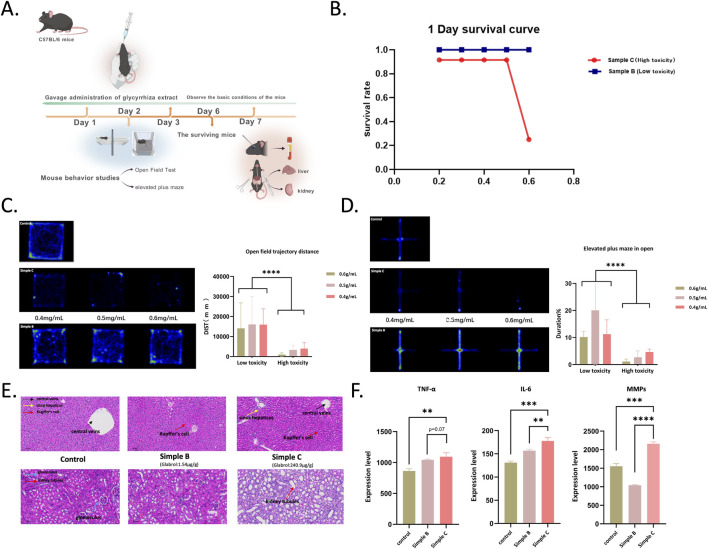
Comparative toxicity assessment of licorice extracts with high (Sample C) and low (Sample B) glabrol content in mice. **(A)** Experimental timeline. Arrows indicate time points of oral gavage. Blood and tissue samples were collected at the end of the gavage period. **(B)** Survival curves of mice following a single oral gavage of licorice extracts with high (Sample C) or low (Sample B) glabrol content. **(C)** Left: Representative heatmaps of mouse movement trajectories in the open field test. Right: Statistical analysis of total movement distance (****p < 0.0001). **(D)** Left: Representative movement traces of mice in the elevated plus maze test. Right: Ratio of time spent in the closed arms to that in the open arms (****p < 0.0001). **(E)** Representative histological sections of liver tissue and kidney tissue from mice after 7 days of gavage with Sample B or Sample C extracts. **(F)** Plasma levels of inflammatory cytokines IL-6, TNF-α, and MMP-9 as determined by ELISA. Data are presented as mean ± SEM (*p < 0.05, **p < 0.01, ***p < 0.001)

To further evaluate the acute toxicity and behavioral effects of glabrol content in licorice extracts on mice, this study selected licorice extract samples with high (Sample C, 319.23 μg/g) and low (Sample B, 1.54 μg/g) glabrol content for gavage administration experiments in mice (Figure 3A). Survival curve analysis (Figure 3B) showed that the mortality rate in the Sample C group was significantly higher than in the Sample B group, with its survival curve declining more rapidly, directly demonstrating that high glabrol content significantly enhances the toxicity of licorice extracts. The Open Field Test (OFT) and Elevated Plus Maze (EPM) were further employed to assess autonomous activity and exploratory behavior in mice. The OFT trajectory heat map (Figure 3C) visually displayed the activity behavior of mice in the high-toxicity group. Compared to the low-toxicity group, the activity range of mice in the high-toxicity group was significantly reduced. Quantitative analysis results further confirmed that the total distance traveled in the OFT was significantly reduced in the Sample C group (*P* < 0.05). The EPM trajectory diagram (Figure 3D) showed that compared to the Sample B group, mice in the Sample C group tended to stay more in the enclosed arms and less in the open arms. Statistical results indicated that the number of entries into the open arms was significantly reduced (*P* < 0.05), and the time spent in the open arms was significantly shorter (*P < 0.05) in the Sample C group. These data collectively indicate that licorice extract with high glabrol content significantly reduced mouse activity.

### 3.5 Effects of glabrol on mouse liver and kidney

Pathological analysis further revealed organ damage in the high-toxicity group mice. In histopathological sections, the livers of mice in the Sample C group showed an increase in Kupffer cells and the appearance of granulomatous hepatitis, while kidney tissues exhibited renal tubular dilation (Figure 3E). Concurrently, plasma levels of inflammatory factors were significantly elevated in the Sample C group, with levels of interleukin-6 (IL-6), tumor necrosis factor-alpha (TNF-α), and matrix metalloproteinases (MMPs) all significantly increased (Figure 3F). Therefore, licorice extract samples with high glabrol content significantly increased mouse mortality, significantly reduced their activity, caused pathological damage to the liver and kidneys, and induced inflammatory responses, indicating that the toxicity and safety of licorice extracts are directly related to their glabrol content.

### 3.6 Exploring the toxicological mechanism of glabrol: transcriptome analysis reveals multi-pathway synergistic effects

**FIGURE 4:**
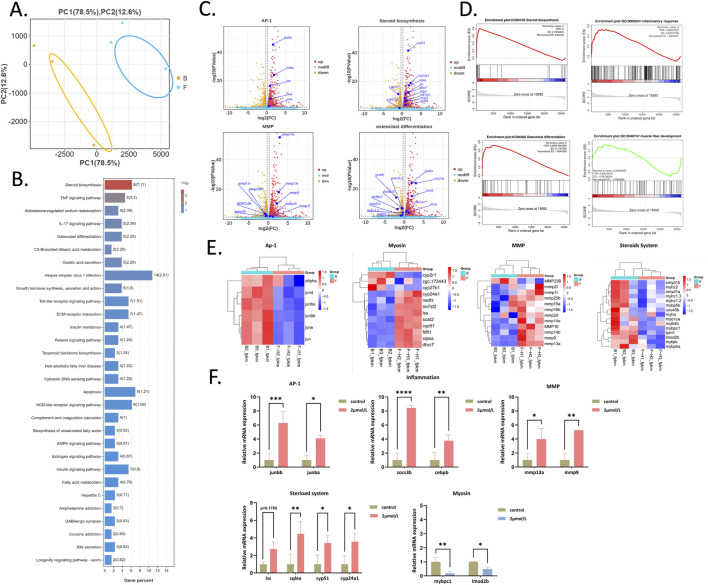
Transcriptomic analysis reveals AP-1 signaling pathway as a key mediator of glabrol toxicity**. (A)**Principal component analysis (PCA) of gene expression profiles from the control and glabrol-treated groups. **(B)** KEGG pathway enrichment analysis. Significantly enriched pathways in the glabrol-treated group include Steroid biosynthesis, Aldosterone-regulated sodium reabsorption, and the TNF signaling pathway. **(C)** Volcano plot displaying differentially expressed genes (DEGs) between glabrol-treated and control groups. Genes with a significant change (|log_2_FC| >1 and *p* < 0.05) are highlighted in red (upregulated) and blue (downregulated). **(D)** Gene Set Enrichment Analysis (GSEA) showing significant enrichment (*p* < 0.05) of gene sets related to aldosterone-regulated sodium reabsorption and steroid biosynthesis in the glabrol-treated group. **(E)** Heatmap visualization of the expression patterns of selected genes involved in the AP-1 signaling pathway, MMP signaling, aldosterone-regulated sodium reabsorption, and muscle generation. Red and blue colors indicate expression levels above and below the mean, respectively. **(F)** RT-qPCR validation of the RNA-seq results. The relative mRNA expression levels of key genes associated with the AP-1 signaling pathway, MMP signaling, muscle generation, and aldosterone-regulated sodium reabsorption were consistent with the transcriptomic data. Data are presented as mean ± SEM (*p < 0.05)

To deeply investigate the toxicological mechanism of glabrol, we conducted comprehensive transcriptome analysis on the glabrol-treated group. Principal Component Analysis (PCA) revealed a clear separation between the glabrol-treated and control groups along the principal component PC1 (Figure 4A). KEGG pathway enrichment analysis results showed significant enrichment of several signaling pathways closely related to toxicological effects, including Steroid biosynthesis, Aldosterone-regulated sodium reabsorption, Osteoclast differentiation, and TNF signaling pathway (Figure 4B). The volcano plot highlighted that genes related to the AP-1 signaling pathway, steroid biosynthesis, MMP activity, and osteoclast differentiation displayed significant alterations in expression (Figure 4C). Gene Set Enrichment Analysis revealed that glabrol treatment significantly perturbed several biological pathways (Figure 4D). Most notably, the “Steroid biosynthesis” pathway was robustly upregulated (NES = 2.007, FDR = 0), accompanied by a significant activation of the “Inflammatory response” (NES = 1.955, FDR <0.001). Another gene set showed a trend of upregulation (NES = 1.653) but did not reach the strict threshold of statistical significance (FDR ≈0.08). In contrast, one gene set exhibited a non-significant downregulation trend (NES = −1.088, FDR = 0.652). These findings indicate that glabrol specifically activates metabolic and inflammatory processes. Further gene expression heatmap analysis revealed the precise regulation of specific gene networks by glabrol (Figure 4E). The study found that glabrol significantly upregulated the expression levels of genes related to the AP-1 signaling pathway. AP-1 (Activator Protein-1) is an important transcription factor complex whose activation is typically associated with cell proliferation, differentiation, apoptosis, and inflammatory responses (Eferl and Wagner, 2003). Regarding downstream effects, we observed that glabrol significantly impacted bone and muscle development. The expression levels of genes related to the matrix metalloproteinase (MMP) pathway (e.g., mmp13a, mmp9) were significantly upregulated. The expression levels of key genes related to myogenesis (e.g., Mybpc-1, Lmod2b) were significantly downregulated. Mybpc-1 (Myosin Binding Protein C) and Lmod2b (Leiomodin 2b) play important roles in the assembly and structural stability of myofibrils; their downregulation directly inhibits normal muscle generation (Wang et al., 2025). Simultaneously, the expression levels of genes related to skeletal differentiation (e.g., Cyp24a1, sqlea, Lss, cyp51) were significantly upregulated, indicating that while inhibiting muscle development, glabrol may promote abnormal bone-related remodeling processes. Additionally, glabrol triggered a strong inflammatory response. The expression levels of genes associated with inflammation generation (e.g., socs3b, cebpb) were significantly upregulated. Socs3b (Suppressor of Cytokine Signaling 3b) plays a role in the negative feedback regulation of inflammatory signaling pathways, and its upregulation may reflect the body’s response to sustained inflammatory signals (Sobah et al., 2023). Cebpb (CCAAT/Enhancer-Binding Protein Beta) is a key transcription factor regulating inflammatory gene expression (Wang et al., 2022). We propose that the coordinated upregulation of these genes is a key event driving the systemic inflammatory cascade observed upon glabrol‘s effects. Therefore, we hypothesize that the toxic mechanism of glabrol may involve the upregulation of the AP-1 signaling pathway as a core regulatory hub, subsequently triggering a series of complex biological effects.

### 3.7 RT-qPCR validates gene expression trends observed in RNA-Seq

To further validate the differentially expressed genes (DEGs) observed in the transcriptome analysis, we selected some representative genes for quantitative analysis using RT-qPCR (Figure 4F). Relative expression levels were normalized to the housekeeping gene gapdh, which showed stable expression across all samples. RT-qPCR results showed that the expression levels of AP-1 signaling pathway-related genes Junba and Junbb, MMP signaling pathway-related genes mmp13a and mmp9, and skeletal differentiation-related genes cyp24a1, sqlea, Lss, and cyp51 were all significantly increased. The expression levels of inflammation-related genes socs3b and cebpb were significantly increased, while the expression levels of key myogenesis-related genes Mybpc-1and Lmod2b were significantly decreased. These results are consistent with the gene expression trends observed in the transcriptome data.

### 3.8 Teratogenic mechanism of glabrol in zebrafish embryos

**FIGURE 5:**
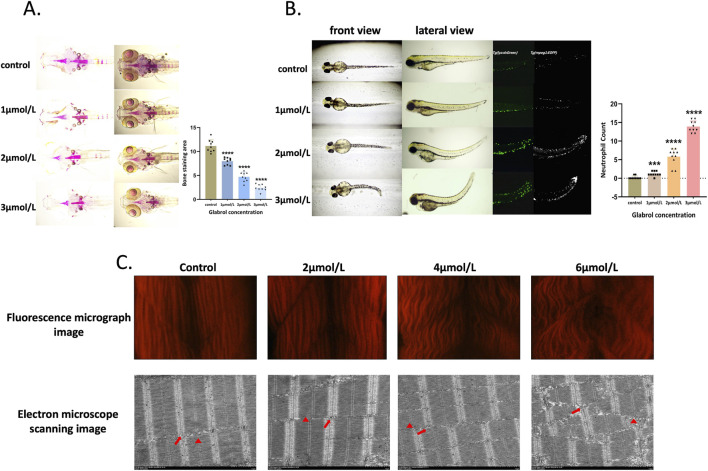
Evaluation of the effects of glabrol on early development and skeletal formation in zebrafish. **(A)** Alizarin Red S staining of bone in zebrafish. The staining reveals the distribution and intensity of mineralized bone structures following glabrol treatment. **(B)** Representative images of wild-type, Tg [mpeg:EGFP], and Tg [lyz:dsGreen] transgenic zebrafish lines treated with glabrol, illustrating its impact on early development. **(C)** The effect of glabrol on myofibril morphology and sarcomere structure in zebrafish embryos. Arrows and triangles indicate the positions of M-lines and Z-lines, respectively, highlighting the structural alterations induced by glabrol exposure.

To further investigate the toxic mechanism of glabrol, zebrafish embryos were treated with the glabrol standard at three different concentrations (1, 2, and 3 μmol/L) and observed for toxicity phenotypes. The results showed that glabrol could cause somite malformations in zebrafish embryos, and the higher the glabrol content, the more pronounced the somite malformations (Figure 5B). After staining the hard bone with Alizarin Red S, we found that the staining intensity was significantly reduced in the glabrol-treated group, indicating inhibited or defective skeletal development (*P* < 0.05) (Figure 5A). Furthermore, through phalloidin staining and transmission electron microscopy, we conducted detailed observations of the muscle tissue in zebrafish embryos (Figure 5C). In the glabrol-treated group, we clearly observed severely disordered arrangement of muscle fibers in the embryonic somites. The degree of disorder intensified with increasing glabrol concentration. By using transgenic fish lines marking neutrophils Tg (lyz:Green) and macrophages Tg (mpeg1:EGFP), we detected that the inflammation level was significantly increased in glabrol-treated zebrafish embryos (*P* < 0.05), further confirming the pro-inflammatory effect of glabrol (Figure 5B). In summary, these experimental results collectively demonstrate that glabrol causes significant teratogenic and toxic effects on zebrafish embryos by affecting muscle fibers, skeletal development, and inducing inflammatory responses.

## 4 Discussion

Licorice is used historically and widely in modern products, leading to substantial human exposure and highlighting the need for rigorous quality control. Current quality assessment methods, which mainly rely on quantifying a limited set of chemical markers, are inadequate for fully evaluating safety and efficacy. Our study, together with existing evidence, suggests that bioactivity evaluation should be incorporated as a necessary complement to chemical analysis. This combined approach not only offers more direct and pharmacologically relevant quality indicators but may also help identify new bioactive compounds, thereby strengthening the overall framework for licorice quality control.

Glabridin is widely considered a safe skin-lightening agent. However, our bioactivity-guided investigation has identified glabrol—a flavonoid from licorice—as a critical toxic impurity that undermines its safety profile (Guo et al., 2022). Previous studies on isolated glabrol reported potential benefits, such as non-competitive inhibition of ACAT and DGAT for anti-atherosclerosis and anti-obesity effects (Choi et al., 2007; Choi et al., 2010) antimicrobial activity against MRSA with antibiotic synergy (Wu et al., 2019; Wang et al., 2024; Liu Y. et al., 2025), and sleep modulation *via* GABAA receptor potentiation (Cho et al., 2012). Yet, the behavior and impact of glabrol within the complex licorice extract milieu remain unexamined.

**FIGURE 6:**
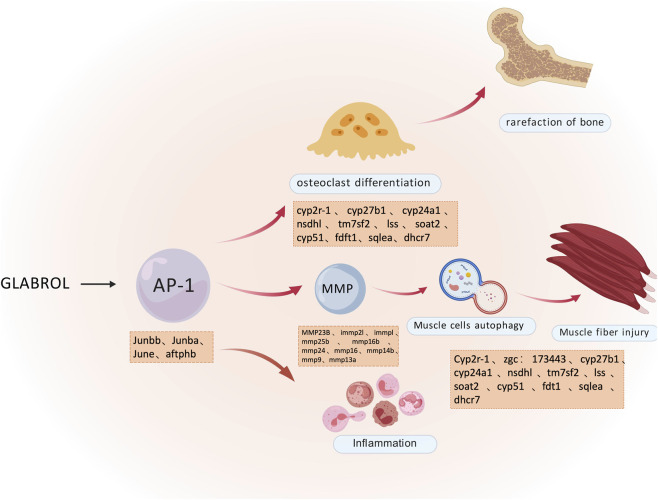
Glabrol exerts toxic effects through the activation of the AP-1 signaling pathway. Schematic representation of the mechanism by which Glabrol induces inflammation and musculoskeletal toxicity *via* the AP-1 signaling pathway.

Our study reveals, for the first time, that glabrol is a ubiquitous impurity in commercial licorice extracts, with its concentration directly correlating with overall toxicity. We further demonstrate that glabrol acts as an upstream signaling activator that upregulates AP-1-associated transcription factors (including Junba and Junbb), forming a central regulatory hub. This core perturbation subsequently modulates downstream effectors involved in MMP activation, osteoclast differentiation, and muscle fiber development, ultimately driving acute toxicity, musculoskeletal damage, and severe inflammatory responses (Figure 6).

Existing research attributes the side effects of long-term or high-dose licorice use, such as hypertension and hypokalemia, traditionally and entirely to the inhibition of 11β-hydroxysteroid dehydrogenase type 2 (11β-HSD2) by glycyrrhizic acid (GL) and glycyrrhetinic acid (GA) (Makino, 2021). However, in our investigation of glabrol, we found that this substance significantly affects the expression of genes related to the steroid pathway. This allows us to reasonably hypothesize that these clinically observed adverse reactions might be partially caused or exacerbated by glabrol. Glabrol could be a key component involved in the classic mechanism of “licorice-induced pseudohyperaldosteronism”. Moreover, clinical reports occasionally note that patients on long-term licorice preparations, alongside the classic symptoms of hypertension and edema, present with rhabdomyolysis, skeletal muscle damage leading to acute renal failure, along with symptoms like muscle weakness, fatigue, and joint pain/cramps. Existing studies often simplistically attribute these symptoms to pseudohyperaldosteronism (Caré et al., 2023; Meltem et al., 2009; McHugh et al., 2021; Caré et al., 2025; Saito et al., 1994; Dandörfer and Studhalter, 2021; Karthik et al., 2025; van den Bosch et al., 2005; Attout et al., 2020). However, our research reveals a novel finding: we have confirmed that glabrol exerts its toxic effects specifically *via* the AP-1 signaling pathway. AP-1 is a well-established master regulator of inflammatory responses (Bejjani et al., 2019; Ji et al., 2019), capable of directly binding to the promoter regions of representative pro-inflammatory cytokines such as TNF-α, IL-6, and IL-1β, driving their expression and causing inflammation (Mishra et al., 2016; Wang et al., 2010; Matsushima et al., 2022; Bhosale et al., 2022; Scheller et al., 2011; Ratajczak-Wrona et al., 2020). Concurrently, the activation of the AP-1 signaling pathway promotes the expression of MMPs (Oh et al., 2024; Vincenti and Brinckerhoff, 2002; Chatterjee et al., 2024). The ensuing dysregulated expression of these enzymes degrades the connective tissue matrix surrounding muscle and bone, leading to a loss of structural integrity and, consequently, functional impairment (Kim and Lee, 2020; Wels et al., 2014; Giannandrea and Parks, 2014; Mehana et al., 2019). Furthermore, our findings, which align with established studies demonstrating a strong association between aberrant AP-1 signaling and skeletal muscle impairment (Ganesan et al., 2017; Almada et al., 2021), lead us to hypothesize that glabrol may contribute to the clinical symptoms of unexplained myalgia and weakness observed with licorice intake by exacerbating AP-1-driven muscle damage.

While our study confirms that glabrol compromises the safety profile of licorice extract, its impact on the extract’s efficacy remains unclear. Existing research indicates that licorice extract can increase dermal collagen by suppressing MMP-1 expression, thereby reducing collagen degradation (Amini et al., 2025). Conversely, our findings demonstrate that glabrol potently activates the AP-1 pathway, upregulating MMPs—the sole enzyme family responsible for collagen catabolism *in vivo*. As MMP overexpression accelerates collagen breakdown, tipping the balance from synthesis to degradation and promoting skin aging (Jabłońska-Trypuć et al., 2016; Pittayapruek et al., 2016), a critical question arises: whether the presence of glabrol mitigates or even reverses the anti-aging benefits of licorice extract. The net effect on collagen homeostasis warrants further investigation.

In summary, while glabrol may not be the exclusive toxicant within the chemically complex matrix of licorice, our study identifies it as a critical risk impurity. The experimental evidence demonstrates that an increase in glabrol content can significantly induce or enhance the toxicity of licorice extracts. Consequently, our findings suggest that glabrol should be incorporated as a key controlled indicator in quality standards for licorice products and strictly regulated. By elucidating the toxicity mechanisms of this potential risk substance, this work provides a crucial scientific basis for ensuring the safe application of licorice extracts.

## Data Availability

The original contributions presented in the study are publicly available. This data can be found here: https://www.ncbi.nlm.nih.gov/sra/PRJNA1439600.
